# Inhibition of T Cell Protein Tyrosine Phosphatase Enhances Interleukin-18-Dependent Hematopoietic Stem Cell Expansion

**DOI:** 10.1002/stem.1276

**Published:** 2012-11-08

**Authors:** Annie Bourdeau, Sébastien Trop, Karen M Doody, Daniel J Dumont, Michel L Tremblayef

**Affiliations:** a Sunnybrook Research InstituteToronto, Ontario, Canada; b Department of Immunology, University of TorontoToronto, Ontario, Canada; c Clinician Investigator Program, University of TorontoToronto, Ontario, Canada; d Division of Cardiac Surgery, St. Michael's HospitalToronto, Ontario, Canada; e Rosalind and Morris Goodman Cancer CentreMontréal, Québec, Canada; f Department of Biochemistry, McGill UniversityMontréal, Québec, Canada; g Department of Medical Biophysics, University of TorontoToronto, Ontario, Canada

**Keywords:** Protein tyrosine phosphatase, Cytokines, Stem cells, Progenitor cells, Small molecule inhibitor, siRNA

## Abstract

The clinical application of hematopoietic progenitor cell-based therapies for the treatment of hematological diseases is hindered by current protocols, which are cumbersome and have limited efficacy to augment the progenitor cell pool. We report that inhibition of T-cell protein tyrosine phosphatase (TC-PTP), an enzyme involved in the regulation of cytokine signaling, through gene knockout results in a ninefold increase in the number of hematopoietic progenitors in murine bone marrow (BM). This effect could be reproduced using a short (48 hours) treatment with a pharmacological inhibitor of TC-PTP in murine BM, as well as in human BM, peripheral blood, and cord blood. We also demonstrate that the ex vivo use of TC-PTP inhibitor only provides a temporary effect on stem cells and did not alter their capacity to reconstitute all hematopoietic components in vivo. We establish that one of the mechanisms whereby inhibition of TC-PTP mediates its effects involves the interleukin-18 (IL-18) signaling pathway, leading to increased production of IL-12 and interferon-gamma by progenitor cells. Together, our results reveal a previously unrecognized role for IL-18 in contributing to the augmentation of the stem cell pool and provide a novel and simple method to rapidly expand progenitor cells from a variety of sources using a pharmacological compound. Stem Cells *2013;31:293–304*

## INTRODUCTION

Techniques to improve cell-based therapies are a clinical imperative to increase the efficacy of chemotherapy and bone marrow (BM) transplantation for the treatment of hematological diseases. Contemporary approaches involve hematopoietic stem cell (HSC) collection and ex vivo expansion with cytokine cocktails. In humans, granulocyte colony stimulating factor, granulocyte-macrophage colony stimulating factor, interleukin-3 (IL-3), erythropoietin, and thrombopoietin are known to increase the number of HSC, individually or in combination [1]. However, current protocols are cumbersome and have limited efficacy, prompting the need for novel therapies that can efficiently expand the progenitor cell pool [2]. This goal may be achieved by developing pharmacological compounds to manipulate the molecular pathways that regulate stem cell/progenitor cell proliferation and responsiveness to cytokines.

Several protein tyrosine phosphatases (PTPs), including T-cell protein tyrosine phosphatase (TC-PTP), have been shown to influence BM HSC, progenitors, and terminally differentiated hematopoietic cells by regulating cytokine signaling [3]. TC-PTP is an intracellular enzyme, encoded by the *Ptpn2* gene, which is ubiquitously expressed but is most abundant in hematopoietic cells. TC-PTP modulates cytokine and growth factor signaling pathways crucial for hematopoietic development and expansion through negative regulation of associated Jak and Stat proteins [4]. The numerous hematopoietic defects observed in TC-PTP-deficient (*tc-ptp*^−/−^) mice, including myeloid, lymphoid, and erythroid lineage defects, reveal the importance of TC-PTP in regulating hematopoiesis and suggest a primary anomaly of HSC [5–8]. Together, these findings imply a role for TC-PTP in the development of the hematopoietic lineages and highlight its potential as a valuable therapeutic target.

In this study, we demonstrate that genetic or pharmacological inhibition of TC-PTP with a RNAi or small molecule inhibitor results in rapid augmentation of BM and circulating HSC. We propose a mechanism whereby inhibition of TC-PTP exerts its effects through modulation of the IL-18/IL-18bp signaling pathway. These results demonstrate the feasibility of using pharmacological manipulation to enhance the efficacy of cell-based therapies.

## MATERIALS AND METHODS

### Mice

*Tc-ptp* and *ptp1b* mutant mice have been described [8, 9]. Experiments were performed with mice on a mixed BALB/c–129SJ background and on mice backcrossed for eight generations on BALB/c. BALB/c mice and BALB/c transgenic mice express enhanced green fluorescent protein under the human ubiqutin C promoter were purchased from Jackson Laboratories, Bar Harbor, ME, http://www.jax.org/. All procedures were approved by the McGill University Research and Ethics Animal Committee or approved by the Sunnybrook Research Institute, Comparative Research Animal Care Committee. All experiments were carried out according to the Canadian Council on Animal Care ethical regulations.

### Flow Cytometry (Murine and Human Cells)

Human BM, peripheral blood (PB), and cord blood (CB) were obtained from StemCell Technologies (Vancouver, BC, Canada, http://www.stemcell.com/). Mouse BM suspensions were prepared from tibia, femur, humerus, and ulna of *tc-ptp*^+/+^ and *tc-ptp*^−/−^ mice (14-19 days), or only from femur of adult BALB/c, *tc-ptp*^+/−^, and *ptp1b*^−/−^ mice (8-12 weeks) in phosphate buffered saline (PBS)/2% fetal bovine serum (FBS). Mouse mononuclear cells were obtained from PB and isolated using Lympholyte M (Cedarlane, Hornby, ON, Canada, http://www.cedarlanelabs.com/), and resuspended in PBS/2% FBS. All murine suspensions were incubated with purified anti-CD16/CD32 mAb (BD Biosciences, Mississauga, ON, Canada, http://www.bdbiosciences.ca), followed by a combination of the indicated antibodies. Both mouse and human flow cytometry reactions were incubated at 4°C for 30 minutes, in 100 μl PBS/2% FBS. Where appropriate, cells were subsequently stained with streptavidin-Pacific Blue at 4°C for 20 minutes, in 100 μl PBS/2% FBS. Data acquisition was performed using a FACSAria or FACS LSRII flow cytometer (BD Biosciences), and analysis was done with FlowJo software (Tree Star, Ashland, OR, http://www.flowjo.com/).

### Proliferation Assay and Mitotic Index

BM or PB were obtained from *tc-ptp*^+/+^ and *tc-ptp*^−/−^ mice (14-19 days) or from adult BALB/c mice (8-12 weeks). Cells (5 × 10^6^) were labeled with 5 μM Carboxyfluorescein diacetate succinimidyl ester CFDA-SE (Invitrogen-Molecular Probes, Burlington, ON, Canada, http://www.invitrogen.com) and cultured for 2 days. Nonadherent cells were stained with the appropriate surface markers and analyzed by flow cytometry. Calculation of the mitotic index was previously described [5].

### Culture Systems: Treatment with RNAi, Small Molecule Inhibitor or IL-18

Mouse BM or a pool of PB cells enriched in progenitor cells (adult BALB/c, *tc-ptp*^+/−^ and *ptp1b*^−/−^ mice, 8-12 weeks; *tc-ptp*^−/−^, 14-20 days); or human BM, PB, and CB cells (Stem Cell Technologies) were used for these in vitro experiments. Cells (5 × 10^6^) were electroporated (320 V, 960 μF) with 1 μM of TC-PTP-specific RNAi (TC1: sense: gcccauaugaucacagucgtt, exon 2, human and mouse) (TC2: sense: ggcacaaagaaguuacauctt, exon 3, mouse only) or scrambled RNAi sequence (SCR). Cells were plated in six-well plates on normal bone marrow stroma, as per our previous manuscript published [5]. Bone marrow stroma was isolated by lineage/stem cell depletion (CD3, CD4, CD5, CD8, CD11b, CD19, DX5, Sca-1, Ter119 cells) using magnetic beads (EasySep, StemCell Technologies) and cultured in Iscove modified Dulbecco media supplemented with 10% FBS and antibiotics. Confluent stromal cells devoid of hematopoietic cells were obtained by serial passage. Experiments were performed using only fresh cells from passage 5 or 6. C-kit^+ and low^ progenitor cells were cultured in StemSpan SFEM media (Stem Cell Technologies) supplemented with 10 μg/ml heparin, 0.53 penicillin/streptomycin, 50 μM 2-mercaptoethanol, 10 ng/ml recombinant mouse SCF, 10 ng/ml Flt3-L, 10 ng/ml IL-11, 30 ng/ml IL-3, 20 ng/ml Tpo, 4 U/ml Epo, 10 ng/ml GM-CSF and with or without serum conditions for 48 hours. For small molecule inhibitor experiments, the inhibitor, described in [10], was added at the start of culture to a final concentration of 10 or 50 μM. Similarly, for the IL-18 stimulation experiments, recombinant mouse IL-18 (R&D Systems, Minneapolis, MN, http://www.rndsystems.com/) or rat anti-mouse IL-18 antibody (93-10C; R&D Systems) was added at the start of the culture. For all the above, cells were harvested after 48 hours of incubation and analyzed by flow cytometry.

### Murine Hematopoietic Stem Cell Transplantation

BALB/c green fluorescent protein (GFP)^+^ donor mouse bone marrow cells were cultured as described above. Forty-eight hours later, GFP^+^Lin^−^CD117^+^Sca-1^+/hi^CD105^+^ stem cells were sorted by flow cytometry using a FACSAria (BD Biosciences). On the same day, the recipient wild-type BALB/c mice were given total body irradiation receiving a total of 8 Gy (800 rd) in two equal split doses of 4 Gy (400 rd; 3 hours apart). Within 4-6 hours after the last irradiation, 100 or 1,000 LT-HSC (GFP^+^LinCD117^+^ Sca-1^+/hi^CD105^+^CD150^+^Flk2^−^) were injected via tail vein in the recipient mice. Weekly starting 7 days post-transplant, 150 μl of peripheral blood was collected from the saphenous vein in a 1 ml heparinized syringe and dispensed in lithium/EDTA tubes on ice. Peripheral blood samples were preserved in 1× lyse/fix reagent according to the manufacturer's instructions (BD Biosciences) and archived at −80°C until analysis. At the time of flow cytometry analysis, lyse/fix blood samples were thawed at 37°C for 10 minutes, cells were collected by centrifugation, washed and resuspended in flow cytometry buffer (PBS and 2% FBS).

### Phosflow and Intracellular Cytokine Staining

Intracellular staining was performed after surface staining. Cells were fixed with 1.5% formaldehyde for 10 minutes at 37°C. Cells were then permeabilized with methanol. The reactions were allowed to stand on ice for 10 minutes. Cells were then washed three times in PBS and resuspended in 100 μl PBS. Cells were blocked and stained with appropriate antibodies for flow cytometry. For phosflow experiments, the mean fluorescence intensity (MFI) was used to evaluate phosphorylation and protein levels. For intracellular cytokine detection, spleen macrophages were used as positive control.

### Statistical Analysis

All statistical differences were determined by two-tailed, unpaired Student's *t* test analysis. The number of samples in each experimental group is indicated in the figure legend of each figure and all data are reported as mean ± SEM.

## RESULTS

### Characterization of Hematopoietic Stem Cells and Progenitor Cells in Tc-ptp^−/−^ Mice

**Figure 1:**
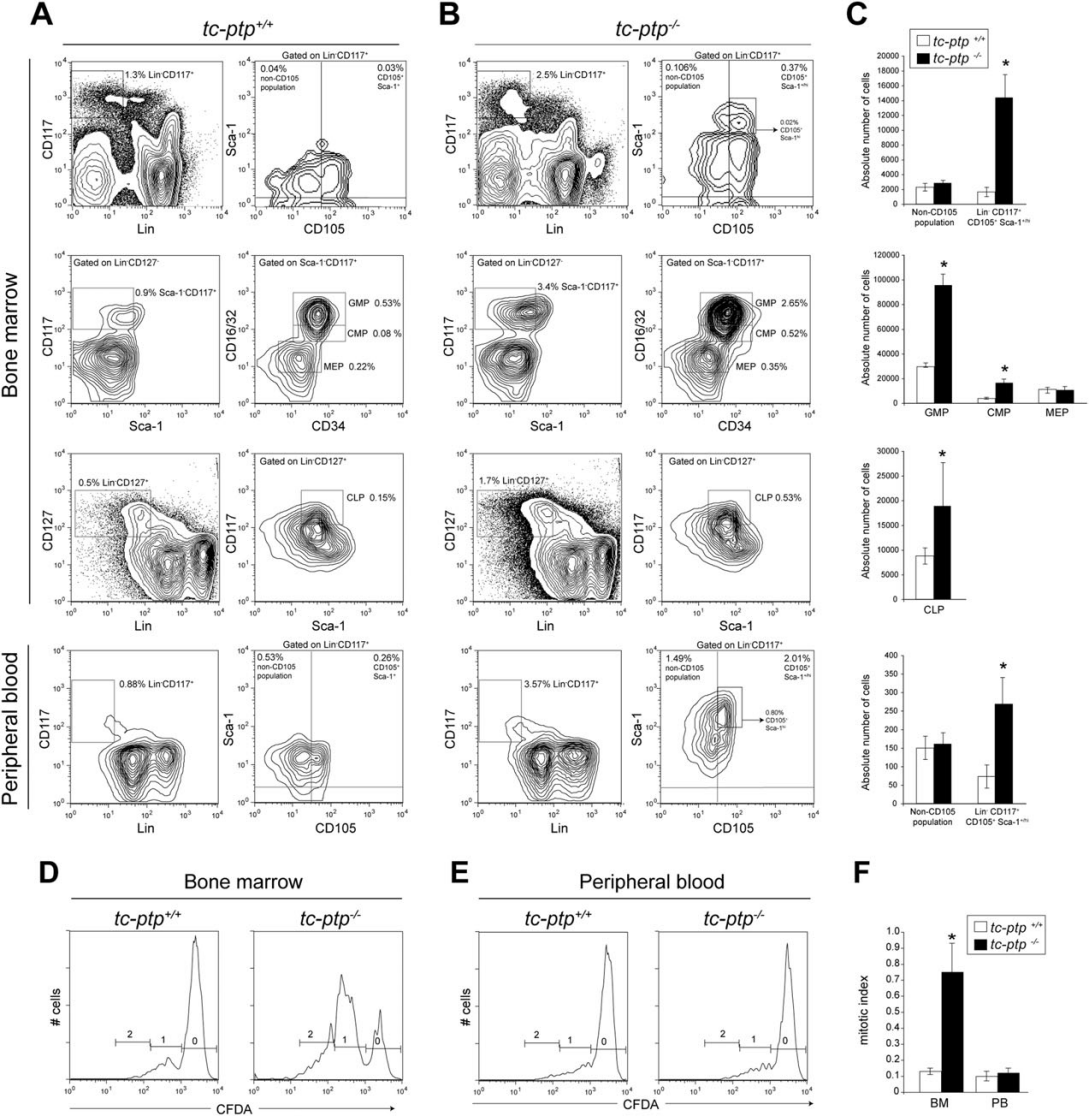
Increased numbers of bone marrow and peripheral blood progenitors in *tc-ptp*^−/−^ mice. **(A,B):***tc-ptp*^+/+^ (panel A) and *tc-ptp*^−/−^ (panel B) BM or PB were stained for stem cells and hematopoietic progenitors and analyzed by flow cytometry. The frequency of each subpopulation is shown. *First row:* BM hematopoietic stem cells (HSCs) were identified as Lin^−^CD117^+^ and according to the expression of Sca-1 (low or high), and CD105. *Second row:* BM CMPs, GMPs, and MEPs were identified as Lin^−^CD127^−^Sca-1^−^CD117^+^ and according to the expression of CD34 and CD16/32. The results shown are gated on the Lin^−^CD127^−^ subpopulation. *Third row:* BM CLPs were identified as Lin^−^CD127^+^ and according to the expression of Sca-1 and CD117. *Fourth row:* PB HSC were identified as Lin^−^CD117^+^ and according to the expression of Sca-1 (low or high) and CD105. **(C):** Absolute cell numbers ± SEM were derived from the relative cell numbers as determined by flow cytometry, and are shown for *tc*-*ptp*^+/+^ (white bars) and *tc-ptp*^−/−^ (black bars) mice. BM subpopulations from *tc-ptp*^+/+^ (*n* = 3) and *tc-ptp*^−/−^ (*n* = 3) mice were analyzed, and samples were read in duplicate; pooled samples of PB were obtained from *tc-ptp*^+/+^ (*n* = 3 pools of 2-3 mice) and *tc-ptp*^−/−^ (*n* = 3 pools of 2-3 mice) animals. *, *p* < .0001 **(D, E):** BM was harvested from *tc-ptp*^+/+^ (*n* = 6) and *tc-ptp*^−/−^ (*n* = 6) mice (panel D), and PB was harvested from *tc-ptp*^+/+^ (*n* = 6 pools of three mice) and *tc-ptp*^−/−^ (*n* = 6 pools of three mice) animals (panel E); cells were labeled with CFDA and cultured for 2 days. Analysis was gated on Lin^−^Sca-1^+/hi^CD117^+^CD105^+^. The number of cell divisions is indicated next to the corresponding CFDA peak. **(F):** The mitotic index for *tc-ptp*^+/+^ (white bars) and *tc-ptp*^−/−^ (black bars) progenitors was calculated from flow cytometry data for BM and for PB, and is reported as mean ± SEM, *, *p* < .001. Abbreviations: BM, bone marrow; CFDA, carboxyfluorescein diacetate succinimidyl ester; CLP, common lymphoid progenitor; CMP, common myeloid progenitor; GMP, granulocyte monocyte progenitor; MEP, megakaryocyte erythrocyte progenitor; PB, peripheral blood.

Hematopoiesis is abnormal in *tc-ptp*^−/−^ mice, resulting in a 35% decrease in BM cellular content [5–8]. To characterize the anomaly in hematopoiesis in these mice, stem cells and progenitor cells obtained from BM of *tc-ptp*^+/+^ and *tc-ptp*^−/−^ mice were analyzed by flow cytometry. HSCs were defined as Lin^−^CD117^+^Sca-1^+^, and this subpopulation was further subdivided based on expression of CD105 (endoglin). CD105 has been identified on subsets of HSC and has been implicated in supporting the differentiation of HSC toward the myeloid and erythroid lineages [11, 12]. We show that it is expressed on long-term HSC (LT-HSC: Lin^−^CD117^+^Sca-1^+^CD105^+^CD150^+^ Flk2^−^) and on subpopulation of short-term HSC (ST-HSC: Lin^−^CD117^+^Sca-1^+^CD105^+^CD150^+^ Flk2^+^) (Supporting Information Fig. 1). In *tc-ptp*^+/+^ BM, two populations were identified: Sca-1^+^CD105^−^ and Sca-1^+^CD105^+^ (Fig. 1A, first row). Two additional populations were observed in *tc-ptp*^−/−^ BM: Sca-1^hi^CD105^−^ and Sca-1^hi^CD105^+^ (Fig. 1B, first row). Moreover, there was a marked increase in the frequency of total BM Sca-1^+/hi^CD105^+^ cells in *tc-ptp*^−/−^ BM (0.37%) compared to *tc-ptp*^+/+^ control (0.03%). Accounting for the decreased cellularity in *tc-ptp*^−/−^ BM, a ninefold increase in the absolute number of Lin^−^CD117^+^Sca-1^+/hi^CD105^+^ cells was observed in *tc-ptp*^−/−^ BM compared to *tc-ptp*^+/+^ control (Fig. 1C, first row). In addition, we have established that *tc-ptp*^−/−^ CD105^+^ HSC had a similar normal apoptotic rate when compared to *tc-ptp*^+/+^ CD105^+^ HSC (Supporting Information Fig. 2). We noted no significant difference in the absolute number of cells in the non-CD105 population (Fig. 1C, first row).

The various BM progenitors derived from HSC were examined in *tc-ptp*^+/+^ and *tc-ptp*^−/−^ mice (Fig. 1A and 1B, second row). The common myeloid progenitor (CMP), which gives rise to granulocyte monocyte progenitors (GMPs) and megakaryocyte erythrocyte progenitors (MEPs), were identified by flow cytometry [13]. A marked increase in the number of cells in all subpopulations was observed in *tc-ptp*^−/−^ BM. However, adjusting for the lower BM cellularity in *tc-ptp*^−/−^ mice, only CMP and GMP populations were truly increased in *tc-ptp*^−/−^ BM, as demonstrated by a three- to fourfold augmentation in absolute cell number (Fig. 1C, second row and [5–8]). The absolute number of common lymphoid progenitors (CLPs) [14] was also augmented 2.5-fold in *tc-ptp*^−/−^ BM compared to *tc-ptp*^+/+^ BM (Fig. 1A, 1B and 1C, third row).

PB Lin^−^CD117^+^Sca-1^+^CD105^+^ stem cells [12, 15] were also quantified (Fig. 1A and 1B; fourth row) to establish if BM HSC were migrating out of the BM. Flow cytometry analysis of pooled blood samples revealed an increase in the total number of PB HSC in *tc-ptp*^−/−^, which translates into a fourfold augmentation of Lin^−^CD117^+^Sca-1^+^CD105^+^ circulating HSC on average, whereas no significant difference in the number of cells in the non-CD105 population was seen (Fig. 1C, fourth row). Thus, the increased number of BM HSC in *tc-ptp*^−/−^ mice is paralleled by a corresponding increase in the number of circulating HSC. To examine the possibility that circulating HSC proliferation could account for their increased number in PB, we compared the proliferation of BM and PB HSC in culture using the cell tracer CFDA. Flow cytometry analysis revealed that >65% of BM progenitors from *tc-ptp*^−/−^ mice had divided once or twice, compared to <20% in *tc-ptp*^+/+^ animals (Fig. 1D). In contrast, *tc-ptp*^−/−^ PB HSC were not proliferating, with ∼80% quiescent, similar to *tc-ptp*^+/+^ PB HSC (Fig. 1E). These results translate into a near sixfold increase in mitotic index between *tc-ptp*^+/+^ and *tc-ptp*^−/−^ BM HSC (0.13 vs. 0.75), whereas PB HSC consistently had a low mitotic index (0.1 vs. 0.12) (Fig. 1F). Hence, the increased number of circulating HSC observed in *tc-ptp*^−/−^ mice most likely reflects their mobilization from the BM. Additionally, analysis of the CFDA profile of *tc-ptp*^−/−^ hematopoietic progenitors (CMP, GMP, and CLP) revealed that these progenitors were not proliferating, suggesting that their increased numbers were most likely due to differentiation of an augmented pool of BM HSC in *tc-ptp*^−/−^ BM (data not shown).

### Effects of TC-PTP-Specific RNAi and Small Molecule Inhibitor on Murine Cells

**Figure 2:**
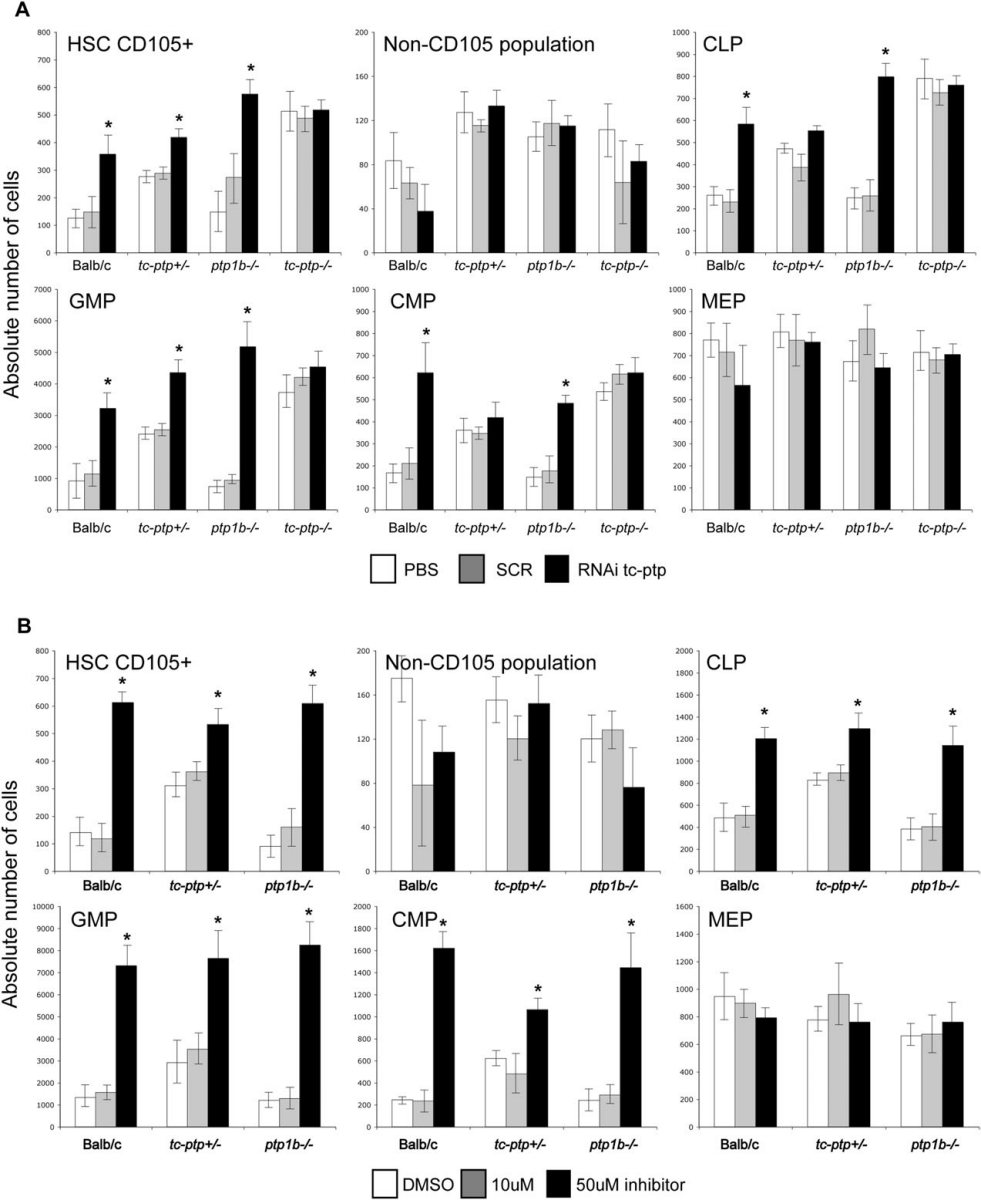
Treatment of murine bone marrow (BM) with TC-PTP inhibitors augments stem cell and progenitor subpopulations. **(A):** BALB/c, *tc-ptp*^+/−^, *ptp1b*^−/−^, and *tc-ptp*^−/−^ BM cells were electroporated without RNAi (PBS; white bars), with a control scramble sequence (SCR; gray bars), or with a pool of TC-PTP RNAi (TC-PTP; black bars). The relative cell number for each BM cell subpopulation was obtained by flow cytometry after 72 hours, and the absolute cell counts ± SEM were calculated for HSC CD105^+^, non-CD105 subpopulation, CLP, GMP, CMP, and MEP. **(B):** BALB/c, *tc-ptp*^+/−^, and *ptp1b*^−/−^ BM were treated with diluent only (DMSO; white bars), with 10 μM small molecule inhibitor (gray bars), or with 50 μM small molecule inhibitor (black bars). The relative cell number for each BM cell subpopulation was obtained by flow cytometry after 48 hours, and the absolute cell counts ± SEM were calculated for HSC CD105^+^, non-CD105 subpopulation, CLP, GMP, CMP, and MEP. BALB/c BM (*n* = 3); *tc-ptp*^+/−^ BM (*n* = 3); *ptp1b*^−/−^ BM (*n* = 3); *tc-ptp*^−/−^ BM (*n* = 3). *, *p* < 0.05. Abbreviations: CLP, common lymphoid progenitor; CMP, common myeloid progenitor; DMSO, dimethyl sulfoxide; GMP, granulocyte monocyte progenitor; HSC, hematopoietic stem cell; MEP, megakaryocyte erythrocyte progenitor; PBS, phosphate buffered saline; SCR, scrambled RNAi sequence.

To confirm that the increased number of stem and progenitors cells observed in *tc-ptp*^−/−^ mice resulted from lack of TC-PTP activity, and not from indirect effects of germline mutation of the *tc-ptp* gene, we used two TC-PTP-specific RNAi, as well as an uncharged thioxothiazolidinone derivative compound [10], to inhibit TC-PTP activity. We suppressed the expression of TC-PTP in BM cultures using a pool of RNAi (Supporting Information Fig. 3A), counted cells and performed flow cytometry analysis of CD105^+^ HSC, non-CD105 population, CLP, GMP, CMP, and MEP to capture the absolute cell number for each population. The average absolute stem cell and progenitor cell counts from several experiments are presented (Fig. 2A). Although not performed under limiting dilution, we observed after 48 hours a two- to fourfold increase in the number of CD105^+^ HSC and progenitor cells (CLP, GMP, and CMP) when BALB/c BM was treated with a pool of TC-PTP RNAi, compared to PBS and scrambled (SCR) RNAi controls. No effect was seen in the non-CD105 population and MEP subpopulation. Similar observations were made in *tc-ptp*^+/−^ BM cultures, although the effect was lesser in magnitude, consistent with reduced expression of TC-PTP in these cells at baseline.

Since TC-PTP shares homology with PTP1B, we examined the possibility that this PTP might also have been affected by treatment with the pool of TC-PTP RNAi. In contrast to *tc-ptp*^−/−^ BM cultures, *ptp1b*^−/−^ BM cultures contained similar numbers of stem cells and progenitor cells compared to wild-type BALB/c BM cultures. Moreover, treatment of *ptp1b*^−/−^ BM with the pool of TC-PTP RNAi resulted in a two- to fivefold increase in the number of CD105^+^ HSC and progenitor cells (CLP, GMP, and CMP) compared to PBS and SCR RNAi controls, similar to the results obtained with wild-type BALB/c BM. Furthermore, no additional effect was seen when *tc-ptp*^−/−^ BM was treated with the pool of RNAi (Fig. 2A). Together, these results indicate that the RNAi were specific for *tc-ptp*.

Unlike RNAi, which must be transfected, small chemical inhibitors possess the advantage of direct uptake by target cells. We identified a unique compound that can penetrate cells and block the catalytic pocket of TC-PTP and PTP1B, leading to increased phosphorylation of their substrates [10]. Since PTP1B does not affect the number of BM progenitors (Fig. 2A and [16]), we reasoned that this compound could be substituted for TC-PTP-specific RNAi. BM cultures from BALB/c, *tc-ptp*^+/−^, and *ptp1b*^−/−^ mice were treated with increasing concentrations of the inhibitor, and the frequency of CD105^+^ HSC, non-CD105 population, CLP, GMP, CMP, and MEP was analyzed by flow cytometry. The average absolute stem and progenitor cell counts from several experiments are presented (Fig. 2B). Treatment of BALB/c BM with inhibitor (50 μM) achieved a 3.5-6-fold increase in the number of CD105^+^ HSC and progenitors cells (CLP, GMP, and CMP) compared to dimethyl sulfoxide (DMSO) control. Treatment of *tc-ptp*^+/−^ BM achieved a smaller (1.6-2-fold) but significant increase in HSC and progenitors, reflecting the reduced expression of TC-PTP in these cells at baseline. Treatment of *ptp1b*^−/−^ BM resulted in a three- to sevenfold augmentation in the number of CD105^+^ HSC and progenitor cells (CLP, GMP, and CMP) compared to DMSO control (Fig. 2B), indicating that this PTP does not influence the number of progenitors. As with RNAi, no effect was seen on non-CD105 population and MEP subpopulation in any of the above cultures.

Together, these results indicate that inhibition of TC-PTP by RNAi or using a small molecule inhibitor may be used to augment stem and progenitor cell numbers identified solely by cell surface markers.

### Effects of TC-PTP-Specific RNAi and Small Molecule Inhibitor on Human Cells

**Figure 3:**
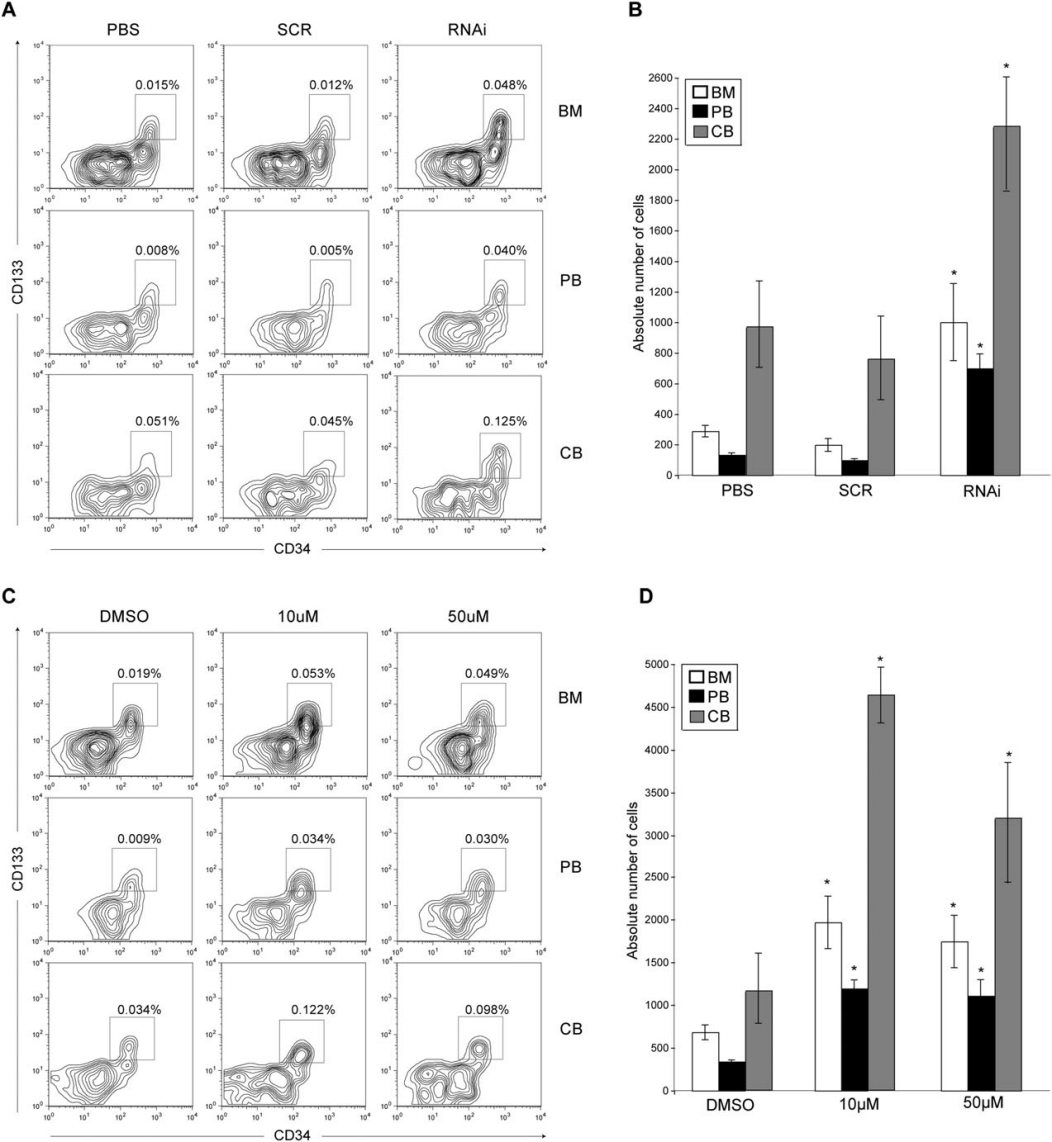
Treatment of human progenitor cells with TC-PTP inhibitors expands the stem cell pool. **(A, B):** Human BM (white bars), PB (black bars) or CB (gray bars) cells were electroporated without RNAi (PBS), with a control SCR, or with TC-PTP RNAi (TC-PTP). The relative cell number for Lin^−^CD34^+^CD133^+^ cells was obtained by flow cytometry after 72 hours, and the absolute cell count ± SEM was calculated (plots are gated on Lin^−^ cells). **(C, D):** Human BM (white bars), PB (black bars) or CB (gray bars) cells were treated with diluent only (DMSO), with 10 μM small molecule inhibitor, or with 50 μM small molecule inhibitor. The relative cell number for Lin^−^CD34^+^CD133^+^ cells was obtained by flow cytometry after 48 hours, and the absolute cell count ± SEM was calculated (plots are gated on Lin^−^ cells). BM (*n* = 3), PB (*n* = 3), and CB (*n* = 4). *, *p* < 0.05. Abbreviations: BM, bone marrow; CB, cord blood; DMSO, dimethyl sulfoxide; PB, peripheral blood; PBS, phosphate buffered saline; SCR, scrambled RNAi sequence.

We tested the capacity of TC-PTP-blocking agents to specifically augment the number of human BM, PB, and CB cells with stem cell properties. Many cell surface markers have been identified that defined population enriched for human HSC. Although heterogenous, the population of cells identified by Lin^−^CD34^+^CD133^+^ has been shown to contain a large proportion of HSC with the highest engraftment capabilities efficiently giving rise to all hematopoietic lineages when transplanted in SCID mice [17, 18]. Human BM, PB, and CB cells were cultured either with TC-PTP-specific RNAi (Supporting Information Fig. 3B) or with small molecule inhibitor. Cells were then counted and the number of Lin^−^CD34^+^CD133^+^ cells was obtained by flow cytometry (Fig. 3A, C). The absolute cell counts calculated from several experiments are shown in Figure 3B and 3D. Although not performed under limiting dilution, the electroporation of TC-PTP RNAi in human BM, PB, and CB cells produced specifically a notable increase in the number of Lin^−^CD34^+^CD133^+^ cells compared to PBS and SCR RNAi controls, which translated into a 3.3-fold (BM), 3.9-fold (PB), and 2.2-fold (CB) increase in the number of stem cells relative to PBS and SCR RNAi controls (Fig. 3A, 3B). Treatment of human cells with the inhibitor (10 μM) resulted in an increased number of all types of Lin^−^CD34^+^CD133^+^ cells and translated into a 3.2-fold (BM), 4.8-fold (PB), and 3.8-fold (CB) increase in the absolute number of Lin^−^CD34^+^CD133^+^ cells relative to DMSO control (Fig. 3C, 3D). There was no significant additional increase in the number of progenitors using a higher concentration of inhibitor of 50 μM (Fig. 3C, 3D). Together, these observations confirm that subpopulations of human stem cells identified based on surface markers can be increased within 48 hours after treatment with either TC-PTP-specific RNAi or small molecule inhibitor compound we have developed, and suggest that pharmacological inhibition of this enzyme might have clinical applicability.

### Evaluation of the Hematopoietic Recovery in Lethally Irradiated Mice Transplanted with Stem Cells Treated with TC-PTP Inhibitor

**Figure 4:**
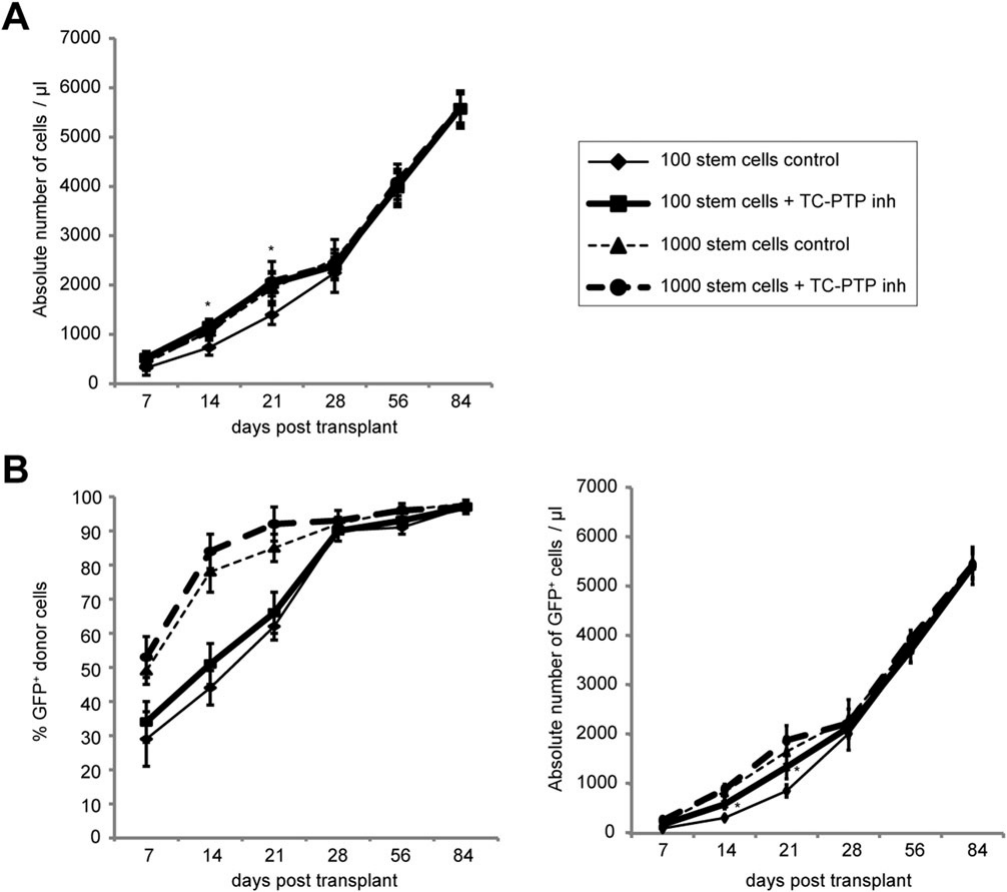
Ex vivo inhibition of TC-PTP does not affect the ability of hematopoietic stem cell (HSC) to reconstitute host animals. Sorted bone marrow HSC CD105^+^GFP^+^ treated with dimethyl sulfoxide (control) or with TC-PTP inhibitor were injected via tail vein (100 or 1,000 stem cells per injection) into lethally irradiated BALB/c mice. Hematopoietic recovery was determined by analysis of peripheral blood samples at the indicated time points. The graphs are representative of five or six mice in each experimental group. **(A):** Peripheral blood absolute mononuclear cell counts per microliter were obtained by adding counting beads to the flow cytometry analysis. **(B):** The percentage of donor-derived cells were determined by number of GFP^+^ cells present in each blood sample. The absolute number of donor-derived GFP^+^ cells were determined also using flowset beads. Absolute cell count or percentage ± SEM was calculated. *, *p* < 0.01. Abbreviations: GFP, green fluorescent protein; TC-PTP, T-cell protein tyrosine phosphatase.

Previous adoptive BM transfer experiments have established that the BM microenvironment of *tc-ptp*^−/−^ mice was unable to support hematopoiesis of *tc-ptp*^+/+^ BM graft. However, *tc-ptp*^−/−^ HSCs are functional and are able to successfully reconstitute normal hematopoiesis in irradiated *tc-ptp*^+/+^ recipients implying that *tc-ptp*^−/−^ HSC production and differentiation was normal in a wild-type BM environment [8]. To confirm that ex vivo use of TC-PTP inhibitor only provides a temporary effect on stem cells and does not alter their capacity to reconstitute all hematopoietic components we cultured for 48 hours BM cells isolated from BALB/c trangenic mice expressing GFP under the human ubiqutin C promoter with 50 μM of TC-PTP inhibitor or DMSO control. Subsequently, 100 or 1,000 Lin^−^CD117^+^Sca-1^+^CD105^+^ cells were injected via tail vein into lethally irradiated nontransgenic BALB/c mice. We performed total peripheral blood white cell counts, total GFP^+^ cell counts (donor origin cells) and multicolor cell subset analyses by flow cytometry beginning 7 days post-transplant. As demonstrated in Figure 4A hosts receiving 100 control stem cells showed a lower absolute number of nucleated cells than hosts receiving 1,000 control stem cells, 14 and 21 days post-transplant (734 cells per microliter vs. 1,057 cells per microliter and 1,396 cells per microliter vs. 1,923 cells per microliter). Mice given 100 TC-PTP treated stem cells had a transiently faster nucleated cell recovery at day 14 and 21 matching the efficiency of the transplant of 1,000 control stem cells (1,169 cells per microliter vs. 1,057 cells per microliter and 2,027 vs. 1,923 cells per microliter). No other differences were noted at any other time points and no differences were noticeable between control and treated groups when 1,000 stem cells were transplanted (Fig. 4A).

This difference in blood nucleated cell counts between 100 TC-PTP-treated stem cell transplants and 100 control stem cell transplants was completely attributed to faster donor-derived cell recovery as shown in both percentage and absolute number of GFP^+^ cells detected in peripheral blood at 14 and 21 days post-transplant (Fig. 4B). These data demonstrate that ex vivo treatment with TC-PTP inhibitor transiently selectively augments donor-derived cell reconstitution in the presence of a limited number of stem cells. We also enumerated the presence of B cells, T cells, and myeloid cells in the peripheral blood samples. We did not find any significant differences in the relative distribution of these subpopulation of cells at any time points (Supporting Information Table 1). These data demonstrate that ex vivo inhibition of TC-PTP only provides a temporary effect and does not impact the ability of HSC to engraft and differentiate into progeny. Importantly, none of the transplanted animals developed hematological malignancies even after 280 days post-transplant.

### Mechanism of Action of TC-PTP Inhibitors

TC-PTP is a negative regulator of cytokine signaling through inhibition of Jak and Stat proteins [4]. *Tc-ptp*^−/−^ mice possess an inflammatory phenotype characterized by increased plasma levels of interferon-gamma (IFN-γ), TNF-α, IL-12, and nitric oxide [6]. Since cytokine signaling pathways regulate HSC proliferation and differentiation, the increase in HSC proliferation observed in *tc-ptp*^−/−^ mice suggests that altered cytokine signaling might underlie the effect of TC-PTP inhibition. Further supporting this notion, microarray analysis of genes expressed in *tc-ptp*^+/+^ and *tc-ptp*^−/−^ CD105^+^ HSC demonstrated differential expression of inflammatory and anti-inflammatory cytokines. Among these, the expression of both IL-18 and IL-18bp was increased twofold in *tc-ptp*^−/−^ progenitors compared to *tc-ptp*^+/+^ progenitors (Bourdeau and Tremblay, unpublished).

**Figure 5:**
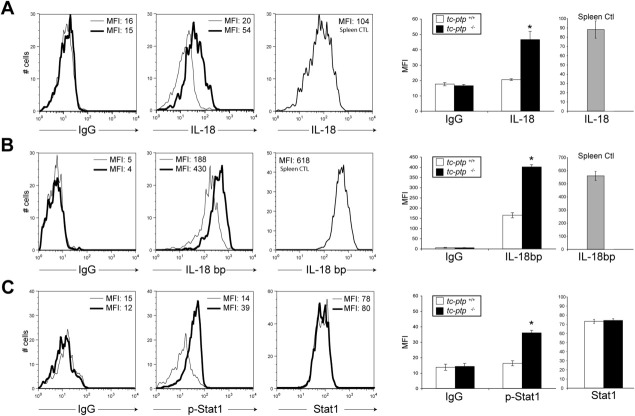
Increased cytokine signaling in *tc-ptp*^−/−^ progenitors. Flow cytometry analysis of bone marrow (BM) hematopoietic stem cells from *tc-ptp*^+/+^ and *tc-ptp*^−/−^ mice was performed to assess intracellular expression of **(A)** IL-18, **(B)** IL-18 binding protein (IL-18bp), or **(C)** phosphorylated Stat1 (p-Stat1) and total Stat1 protein. Analysis was gated on BM Lin^−^Sca-1^+/hi^CD117^+^CD105^+^ cells. Staining of *tc-ptp*^+/+^ splenocytes was performed as positive control (A, B: third flow cytometry histogram). Nonspecific IgG was used as negative control (A, B, C: first flow cytometry histogram). The relative cell number is plotted against IgG, IL-18, IL-18bp, p-Stat1 or Stat1 fluorescence (thin line: *tc-ptp*^+/+^ BM or spleen; thick line: *tc-ptp*^−/−^ BM). The MFI is indicated in the corresponding plot and presented in graphic format (white bars: *tc-ptp*^+/+^ BM, *n* = 4; black bars: *tc-ptp*^−/−^ BM, *n* = 4; gray bars: *tc-ptp*^+/+^ spleen positive control, *n* = 2). Data are provided as MFI ± SEM. *, *p* < 0.05. Abbreviations: IL, interleukin; MFI, mean fluorescence intensity.

Accordingly, we assessed cytokine expression and signaling in *tc-ptp*^−/−^ CD105^+^ HSC. Due to the rarity of these cells, we used phosflow technology for intracellular cytokine and Stat protein detection by flow cytometry. Intracellular levels of IL-18 were higher in *tc-ptp*^−/−^ progenitors compared to *tc-ptp*^+/+^ progenitors, as demonstrated by a twofold shift in MFI (Fig. 5A). In response to IL-18 production, IL-18 binding protein (IL-18bp) and other cytokines are induced [19]. Levels of IL-18bp were elevated in *tc-ptp*^−/−^ CD105^+^ HSC, as indicated by a 2.5-fold increase in MFI (Fig. 5B). Spleen macrophages were used as positive control for the detection of intracellular IL-18 and IL-18bp (Fig. 5A,B). Stat1 becomes phosphorylated upon stimulation with several inflammatory cytokines, including IL-18 [20, 21]. CD105^+^ HSC from *tc-ptp*^−/−^ mice showed a 2.1-fold increase in Stat1 phosphorylation compared to *tc-ptp*^+/+^ animals. Equal amounts of Stat1 protein were detected in both groups (Fig. 5C). Dissecting this phenotype further reveals that the highest levels of intracellular IL-18, IL-18bp, and phospho-Stat1 (p-Stat1) are within the *tc-ptp*^−/−^ Lin^−^CD117^+^Sca-1^hi^CD105^+^ subpopulation based on MFI obtained by flow cytometry (Supporting Information Fig. 4). The expression and activation of other Stat family members were similar between *tc-ptp*^−/−^ and *tc-ptp*^+/+^ HSC. These data suggest that CD105^+^ HSC can secrete IL-18 and activate Stat1-dependent signaling, and induce compensatory IL-18bp expression.

**Figure 6:**
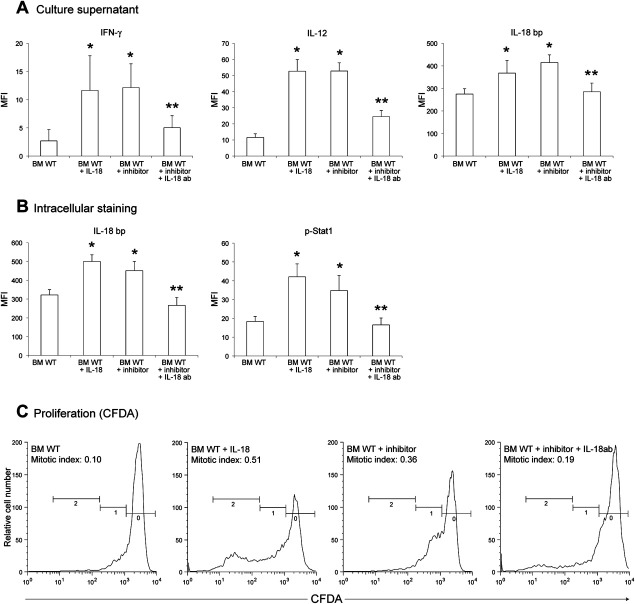
Inhibition of TC-PTP stimulates bone marrow (BM) hematopoietic stem cell expansion through the IL-18 signaling pathway. **(A,B):** BM was harvested from 7- to 10-week old BALB/c mice. Cells were cultured for 48 hours alone (BM WT; *n* = 3), or with 10 μg/mL IL-18 (BM WT + IL-18; *n* = 3), 50 μM TC-PTP inhibitor (BM WT + inhibitor; *n* = 3), or 50 μM inhibitor and IL-18 capture antibody (BM WT + inhibitor + IL-18 ab; *n* = 3). The experiment was repeated three times and each bar graph represents the average MFI obtained by flow cytometry analysis for each group. Data are provided as MFI ± SEM. *, *p* < .05 when compared to BM WT and **, *p* < .05 when compared to BM WT + IL-18 or BM WT + inhibitor. (A) Culture supernatants were compared by flow cytometry for relative content of IFN-γ, IL-12, and IL-18bp. (B) Combined surface and intracellular staining was used to assess intracellular IL-18bp and p-Stat1 expression in hematopoietic stem cells (Lin^−^Sca-1^+/hi^CD117^+^CD105^+^). **(C):** BM from each experimental group was labeled with the cell tracer CFDA and cultured for 48 hours as described above. Hematopoietic stem cells (Lin^−^Sca-1^+/hi^CD117^+^CD105^+^) were then analyzed by flow cytometry. Relative cell number is plotted against CFDA fluorescence. The number of cell divisions is indicated next to the corresponding CFDA peak and the mitotic index is provided. The flow cytometry CFDA plots shown are representative of three independent experiments each group was assessed in triplicate. Abbreviations: BM WT, wild-type bone marrow; CFDA, carboxyfluorescein diacetate succinimidyl ester; IFN-γ, interferon-gamma; IL, interleukin; MFI, mean fluorescence intensity.

Next, we tested the ability of IL-18 to regulate HSC proliferation in our model by manipulating the IL-18/IL-18bp cascade in vitro. Culture of wild-type BM in the presence of IL-18 initiated significant changes in the production of IFN-γ, IL-12, and IL-18bp by CD105^+^ HSC (Fig. 6A,B), activation of Stat1 (Fig. 6B), and ultimately increased their proliferation (Fig. 6C). Thus, treatment with IL-18 recapitulated the effect of TC-PTP inhibitor on wild-type BM (Fig. 6). Confirming the requirement for IL-18 in mediating the effect of TC-PTP inhibition, the addition of IL-18 capture antibody to BM cultures treated with TC-PTP inhibitor suppressed the secretion of IFN-γ and IL-12, and returned soluble IL-18bp to normal levels (Fig. 6A). Intracellular IL-18bp and p-Stat1 levels also returned to baseline (Fig. 6B). Concomitantly, addition of IL-18 antibody mitigated the proliferation of CD105^+^ HSC, as measured by CFDA content by flow cytometry (Fig. 6C).

Together, these results indicate that inhibition of TC-PTP can result in increased IL-18 secretion by CD105^+^ HSC, which in turn promotes activation of the Stat1 cytokine signaling pathway and can increase the production of the counter-regulatory IL-18bp. These findings highlight a role for the IL-18/IL-18bp signaling axis in HSC proliferation.

## DISCUSSION

We report that inhibition of TC-PTP activity through gene knockout or through transient pharmacological inhibition with RNAi or small molecule inhibitor produces significant augmentation of the number of CD105^+^ HSC in mouse BM. The number of circulating HSC is also increased, as well as the number of CMP, GMP, and CLP progenitors in the BM. These observations could be reproduced in various mouse and human stem/progenitor subpopulations in vitro, where significant increase in their number could be observed within 48 hours. Inhibition of TC-PTP in mouse CD105^+^ HSC was associated with increased secretion of IL-18 and activation of Stat1, as well as production of IFN-γ, IL-12, and IL-18bp. These findings reveal a role for the IL-18/IL-18bp axis in contributing to the increased number of HSC.

Previous adoptive BM transfer experiments have established that BM microenvironment of *tc-ptp*^−/−^ mice was unable to support hematopoiesis of *tc-ptp*^+/+^ BM graft. However, *tc-ptp*^−/−^ HSCs are functional and are able to successfully reconstitute normal hematopoiesis in irradiated *tc-ptp*^+/+^ recipients. These transplanted mice contained T and B lymphocytes, and the number and appearance of lymph nodes and spleen was similar to control animals. The hematocrit was likewise normal, indicating reconstitution of both lymphoid and erythroid lineages. The myeloid lineage was not assessed in these experiments; however, *tc-ptp*^−/−^ mice contain monocytes, macrophages, and all granulocyte populations. Importantly, none of the transplanted animals developed hematological malignancies [8]. These data demonstrate that the hematopoietic defects seen in *tc-ptp*^−/−^ mice are rather due to BM stroma defects rather than an autonomous hematopoietic cellular defect [5, 8]. These data are also in agreement with our current hematopoietic reconstitution experiment results where the ability of HSC to engraft and to differentiate into functional progeny is not affected. However, differences in results could be possible as we compared the effects of an irreversible gene deletion (*tc-ptp*^−/−^) in mice to a temporary effect seen with a 48 hours ex vivo treatment of cells with a TC-PTP small molecule inhibitor, which are two different methods that can lead to discrepancies.

Within the CD105^+^ HSC pool, two populations could be distinguished based on surface expression of Sca-1. In *tc-ptp*^−/−^ mice, Sca-1^hi^ HSC represented approximately 5% of the total Sca-1^+^ HSC pool; this HSC population could not be detected in wild-type mice. The most likely explanation for this difference is that the number of Sca-1^+^ HSC was increased ninefold in *tc-ptp*^−/−^ mice compared to control, allowing detection of the rare population of Sca-1^hi^ HSC. Lin^−^CD117^+^Sca-1^hi^ HSC have previously been identified during fetal hematopoiesis [22], and this subpopulation of HSC was shown to contain progenitors that could give rise to all hematopoietic lineages. Lin^−^CD117^+^Sca-1^hi^ HSC have also been described in adult BM [23]. Sca-1 expression is associated with a more immature progenitor phenotype but has not been associated with a developmental block. Conversely, downregulation of Sca-1 expression is associated with differentiation of HSC into myeloid progenitor cells; similarly, CLP retain expression of Sca-1, but their progeny lose expression of this marker [24, 25]. Together, these results indicate that inhibition of TC-PTP leads to increased number of early HSC, prior to lineage commitment. In addition, it has been suggested that heterogeneity of a clonal stem cell population based on surface expression of Sca-1 exists and may be implicated in cell fate decision in stem cells [26]. Our data presented in Supporting Information Figure 4 support that the *tc-ptp*^−/−^ Lin^−^CD117^+^Sca-1^hi^ CD105^+^ stem cell subpopulation have distinct biological function. It has been shown that Sca-1^hi^ cells contain low GATA1 and high PU.1 mRNA favoring their differentiation into cells of the myeloid lineage as opposed to the differentiation into cells of the erythroid lineage as compared to Sca-1^lo^ or Sca-1^mid^ cells [26]. GATA1 and PU.1 suppress each other's activity by direct protein interaction and favor changes in lineage fate [27]. Thus, decreasing TC-PTP activity may favor differentiation into cells of the myeloid lineage and would correlate with the described role of TC-PTP as a negative regulator of CSF-1 signaling leading to increased numbers of macrophages in *tc-ptp*^−/−^ mice [7].

**Figure 7:**
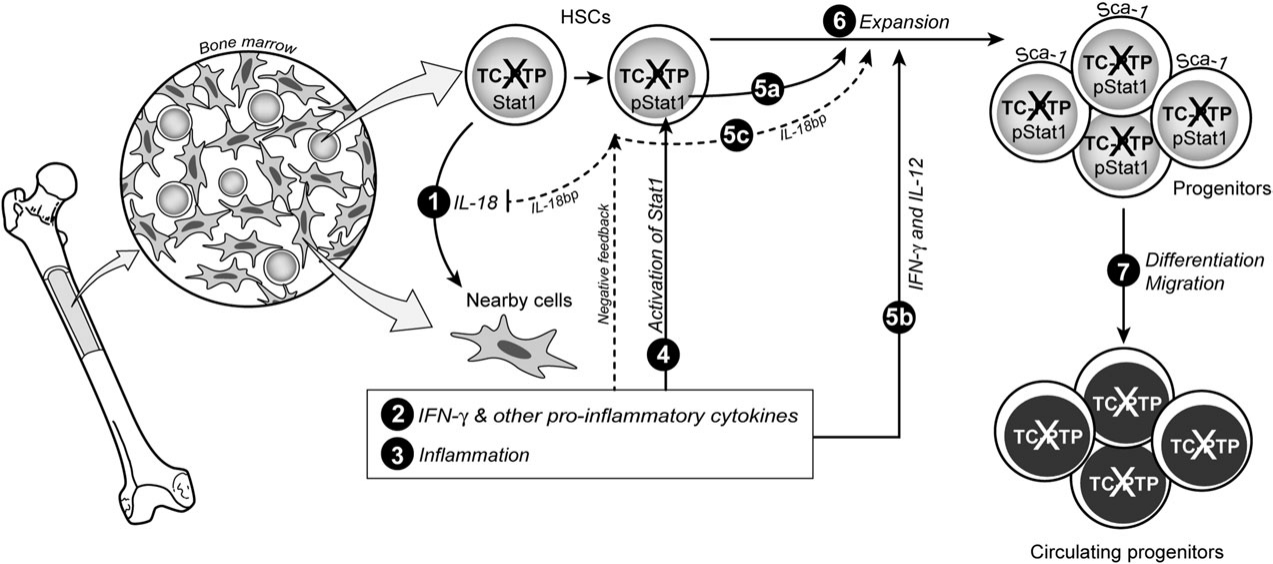
Proposed model of bone marrow (BM) hematopoietic stem cell expansion by the inhibition of TC-PTP **(1):** In the absence of TC-PTP, HSCs in the BM secrete high amounts of IL-18. **(2):** IL-18 induces the secretion of IFN-γ and other pro-inflammatory cytokines by nearby cells. **(3):** This creates a local inflammation **(4)** leading to activation of Stat1. **(5a):** The Stat1 signaling pathway can induce the expansion of HSC. **(5b):** The increased production IFN-γ and other pro-inflammatory cytokines such as IL-12 further contribute to the expansion of HSC. **(6):** The cytokine milieu also induces increased expression of Sca-1 on the surface of HSC. The localized and temporary inflammatory state triggers a negative feedback mechanism to the HSC and induces the production of IL-18 binding protein (IL-18bp). **(5c):** The presence of IL-18bp and cytokines expands the number of HSC and **(7)** facilitates their migration to the periphery. Abbreviations: HSC, hematopoietic stem cell; IFN-γ, interferon-gamma; IL, interleukin.

Upon inhibition of TC-PTP, HSC secrete increased amounts of IL-18 and Stat1 phosphorylation is augmented in these cells, suggesting that this PTP affects HSC activity through regulation of cytokine signaling. This is consistent with previous reports demonstrating that the Jak and Stat families of second messengers are substrates for TC-PTP [4]. These results provide the first evidence that IL-18 is important for regulating HSC biology. This finding integrates well with our current understanding of cytokine signaling in HSC. Indeed, IL-18 has previously been shown to induce production of IL-12 and IFN-γ in hematopoietic and nonhematopoietic cells alike [28-30], which both enhance HSC expansion. In sublethally irradiated mice, injection of IL-12 was found to result in robust recovery of hematopoiesis, which was associated with expansion of Sca-1^+^ BM progenitor cells [31]. IFN-γ is sufficient to expand long-term repopulating HSC in vivo, and activates quiescent HSC [32]. In addition, defective proliferation of HSC has been observed in IFN-γ^−/−^ mice, highlighting the role of this cytokine in regulating HSC activity. Stimulation of Lin^−^CD117^+^ HSC with IFN-γ results in increased expression of Sca-1 on these cells, a process that is dependent on the Stat1 signaling pathway [33]. Stat1 also becomes phosphorylated upon stimulation with several inflammatory cytokines, including IL-18 [20, 21]. Stat1 activation can regulate the proliferation of HSC [34] as well as a variety of progenitor cells such as myoblasts and renal progenitors [35, 36]. We previously reported that the BM microenvironment in *tc-ptp*^−/−^ mice is enriched in IL-12 and IFN-γ [5, 6]. From the above, we propose one of the mechanisms underlying the effects of TC-PTP inhibition in HSC as depicted in Figure 7. In the absence of TC-PTP, HSC in the BM secrete high amounts of IL-18. IL-18 can induce the secretion of IFN-γ and other proinflammatory cytokines by nearby cells. This creates a local inflammation leading to activation of Stat1. The Stat1 signaling pathway and increased production of IL-12 and IFN-γ, further contribute to expansion of HSC and increases expression of Sca-1 on the surface of HSC. The cytokine milieu triggers a negative feedback mechanism to the HSC and induces the production of IL-18bp. The presence of IL-18bp and cytokines expands the number of HSC and facilitates their migration to the periphery. We have not tested the effects of inhibition of TC-PTP in IL-18^−/−^ mice, which are phenotypically normal and fertile but produce no IL-18, since these animal have decreased IFN-γ but can produce normal levels of IL-12 we could not exclude the possibility of the direct effect of the inhibitor on the IL-12 signaling pathway [37]. The model presented in Figure 7 is valid in the context of the *tc-ptp*^−/−^ mice BM microenvironment, but we also think that a similar mechanism occurs when HSC are treated ex vivo but with the benefit that this treatment is only temporary in the HSC. Thus, upon RNAi treatment or pharmacological inhibition of TC-PTP, progenitors are only transiently reprogrammed to enhance their own activity (IL-18, IFN-γ, IL-12, and IL-18bp secretion), through altered cytokine signaling via activation of Stat1.

The ability to rapidly expand a functional population of HSC through pharmacological inhibition of TC-PTP represents a significant finding of this study. The capacity to augment a pool of stem cells is clinically relevant, particularly for the treatment of hematological diseases. Multiple clinical studies in this field have demonstrated the feasibility of expanding BM, circulating or placental progenitors ex vivo using a variety of cytokine and growth factor cocktails [2]. However, each of these studies has been hindered by a number of factors, including inefficient progenitor expansion, exhaustion of the stem cell pool owing to culture conditions, lengthy incubation periods (8-12 days) limiting clinical applicability, or expansion limited to a subpopulation of the progenitor pool. In contrast, we have found that a small molecule inhibitor of TC-PTP or a specific RNAi against TC-PTP could produce rapid (within 48 hours) and significant (ninefold) expansion of functional early stem cells, leading to increased numbers of multiple progenitor populations, including CMP, GMP and CLP. This simple method further avoids the need for genetic alterations, another approach which although efficacious, entails more complex manipulations. Moreover, this technique seems to only provide a temporary effect on stem cells and was successful for stem cells derived from BM, PB as well as CB, providing a broad range of potential clinical uses. The ability to expand CB stem cells may be particularly advantageous given that the use of these cells is rapidly becoming the standard of care, or at least an alternative, for the treatment of many hematological disorders and malignancies (reviewed in [38]). The use of CB is also being investigated for the treatment of many other diseases, such as type 1 diabetes mellitus, spinal cord injury, and inherited biochemical diseases to name a few. Because of the transient effect on stem cells, it is very unlikely that this would lead to stem cell exhaustion; however, serial transplant experiments in animals would be required to sustain these claims. Whether treatment of stem cells with TC-PTP inhibitor or RNAi also expands the pool of progenitors with nonhematological tissue regenerative properties or whether the concurrent inflammatory state or genetic background of the donor or recipient patients (reviewed in [39]) will influence the efficiency of the HSC expansion also remains to be determined. Despite these outstanding questions, we demonstrated several favorable characteristics that would support the potential clinical applicability of using three different and complementary approaches to inhibit TC-PTP for enhancing cell-based therapies.

## CONCLUSION

This study advances the field of stem cell biology by expanding our knowledge of the role of PTP in regulating HSC activity, and by uncovering a role for IL-18 in contributing to the increased number of these cells. The demonstration that with three different approaches: a genetic germline knockout, a RNAi downregulation or a simple pharmacological intervention can achieve significant augmentation of the BM stem cells provides a novel avenue to improve cell-based therapies. Further studies will be directed at confirming the safety and feasibility of some of these promising concepts.

## Supplementary material

Supplemental Figure 1. Characterization of bone marrow hematopoietic stem cells. Further characterization of tc-ptp+/+ Lin-CD117+Sca-1+CD105+ and Lin-CD117+Sca-1+CD105- bone marrow stem cells subpopulations by flow cytometry showing expression of CD150, Flk-2 and CD34.

Supplemental Figure 2. Assessment of apoptosis in hematopoietic stem cells. tc-ptp+/+ and tc-ptp−/− bone marrow (BM) were stained for hematopoietic stem cells as well as Annexin V and propidium iodide (PI) for analyzed by flow cytometry. The results shown are represented as Annexin V and PI and are gated on BM hematopoietic stem cells (HSC), which were identified as Lin-CD117+Sca-1+CD105+ cells.

Supplemental Figure 3. Inhibition of tc-ptp by RNAi treatment. Mouse and human hematopoietic stem cells were electroporated without RNAi (PBS), with a control scramble sequence (SCR), or with TC-PTP RNAi (TC1 and/or TC2). RNA was isolated 72h post treatment from (A) Lin−CD117+Sca-1+CD105+ hematopoietic stem cells from the bone marrow of Balb/c mice and (B) Lin-CD34+CD133+ hematopoietic stem cells from human bone marrow. RT-PCR was performed to analyze levels of TC-PTP. GAPDH was used as a loading control.

Supplemental Figure 4. Further increased cytokine signaling in tc-ptp−/− Lin−CD117+Sca-1hi CD105+ subpopulation. Flow cytometry analysis of bone marrow Lin−CD117+Sca-1+ CD105+ cells from tc-ptp+/+ mice (thin line), tc-ptp−/− Lin−CD117+Sca-1+/hiCD105+ (thick line) and from tc-ptp−/− Lin−CD117+Sca-1hiCD105+ (extra-thick line) was performed to assess intracellular expression of (A) IL-18, (B) IL-18 binding protein (IL-18bp), or (C) phosphorylated Stat1 (p- Stat1) and total Stat1 protein. To better compare subpopulations, both the thin and thick line in all histograms have been reproduced as shown in Figure 5. A further overlay of the tc-ptp−/− Lin− CD117+Sca-1hiCD105+ (extra-thick line) population was performed. Nonspecific IgG was used as negative control (A, B, C: first flow cytometry histogram). The relative cell number, expressed as % max, is plotted against IgG, IL-18, IL-18bp, p-Stat1 or Stat1 fluorescence to compensate for the difference in cellularity between the populations. The mean fluorescence intensity (MFI) is indicated in the corresponding plot.
