# Supplementary figures and images for: Inhibition of T Cell Protein Tyrosine Phosphatase Enhances Interleukin-18-Dependent Hematopoietic Stem Cell Expansion
Source: Stem Cells. 2012 Nov 8;31(2):293–304. doi: 10.1002/stem.1276 (PMC3593175; doi:10.1002/stem.1276)

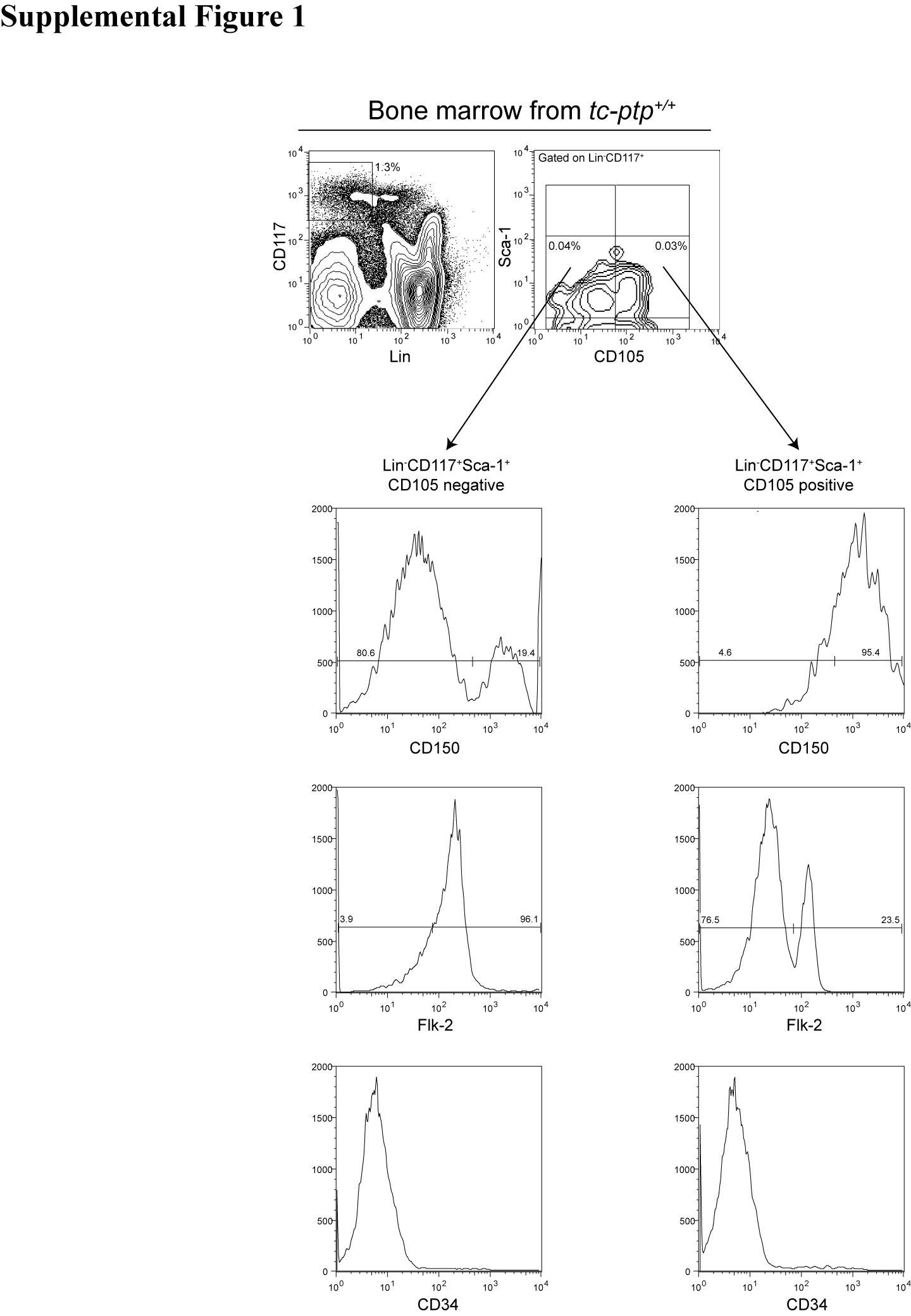

Supplement: Supplementary file 1 [file stem0031-0293-SD1.tif]

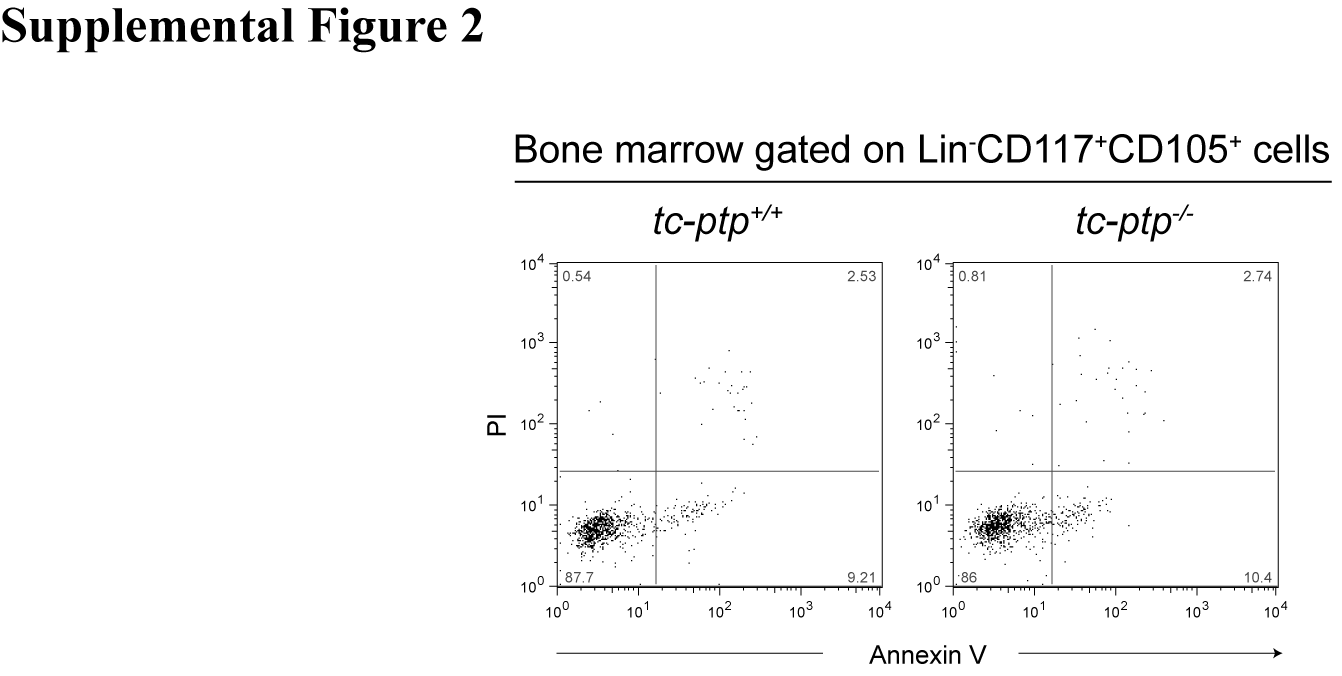

Supplement: Supplementary file 2 [file stem0031-0293-SD2.tif]

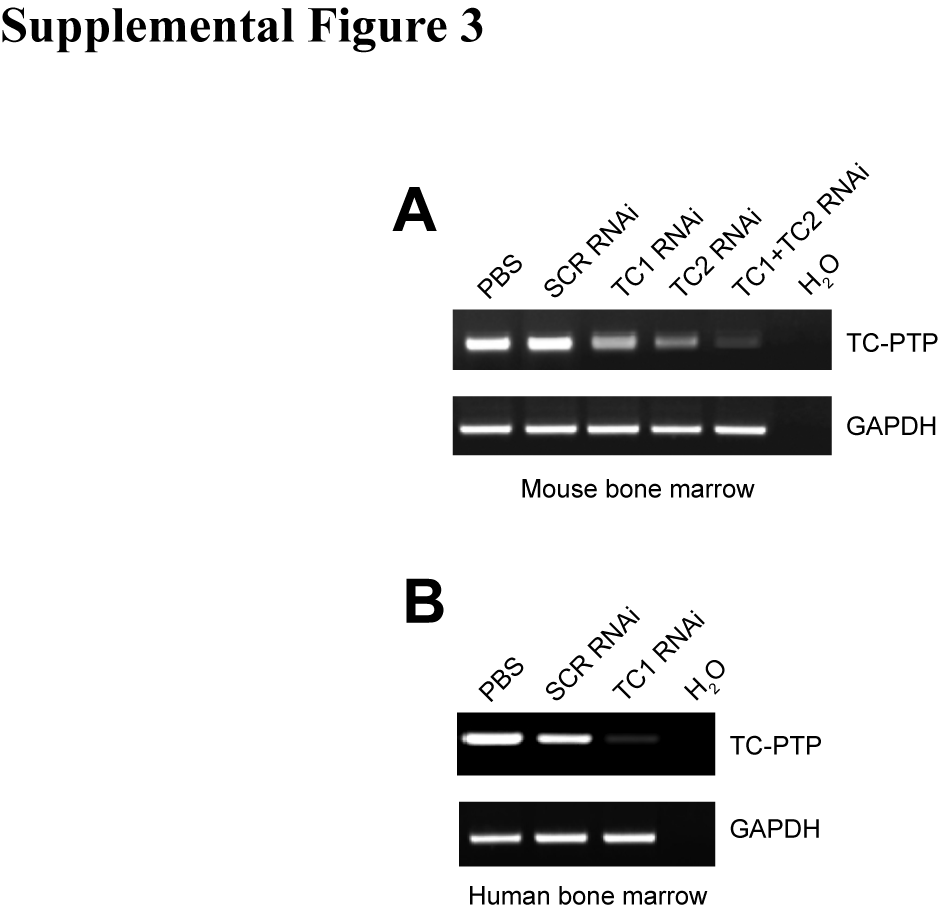

Supplement: Supplementary file 3 [file stem0031-0293-SD3.tif]

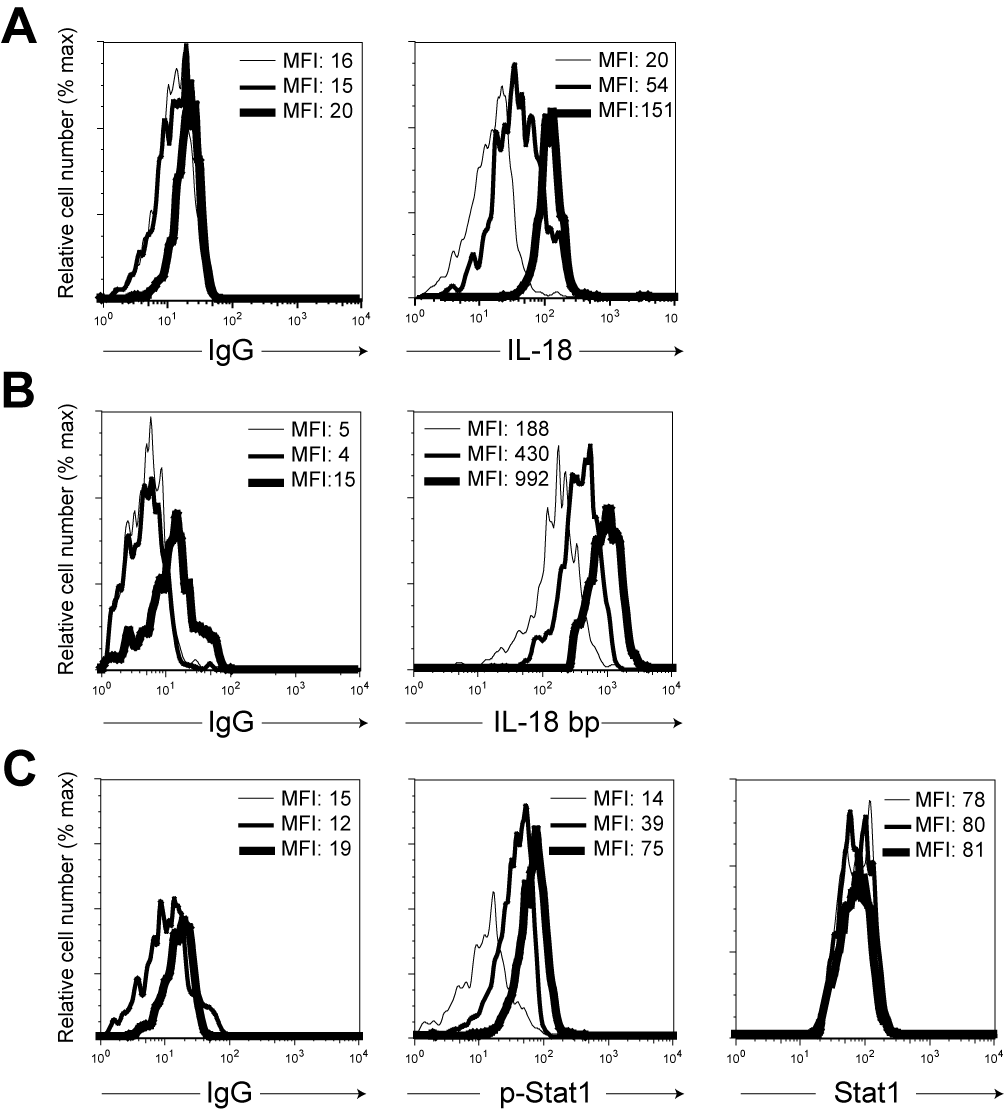

Supplement: Supplementary file 4 [file stem0031-0293-SD4.tif]
