# Supplementary material for: Inhibition of T Cell Protein Tyrosine Phosphatase Enhances Interleukin-18-Dependent Hematopoietic Stem Cell Expansion
Source: Stem Cells. 2012 Nov 8;31(2):293–304. doi: 10.1002/stem.1276 (PMC3593175; doi:10.1002/stem.1276)
Supplement: Supplementary file 5 [file stem0031-0293-SD5.pdf]

Mouse antibodies used

Antibody-fluorochrome conjugates used were: CD105-biotin (MJ7/18; eBioscience, San Diego, CA) CD117-PECy5 or CD117-APC (2B8; Biolegend, Vineland, ON, Canada), Sca-1-FITC or Sca-1 PECy7 (D7; BD Biosciences, Mississauga, ON, Canada), CD127-PE (SB/199; BD Biosciences), CD16/32 FITC (2.4G2; BD Biosciences) CD34-APC (RAM34; BD Biosciences) streptavidin-Pacific Blue (Molecular Probes, Burlington, ON, Canada). Lineage (Lin) markers comprised the following combination of antibody-PE or PE-Cy5 conjugates: CD3ε (145-2C11; BD Biosciences), CD4 (RM4-5; BD Biosciences), CD5 (53-7.3; Biolegend), CD8 (53-6.7; BD Biosciences), CD11b (M1-70; BD Biosciences), CD19 (1D3; BD Biosciences), CD49d (9C10; Biolegend), Ter-119 (Ter119; BD Biosciences). For phosflow and intracellular or supernatant cytokine detection: anti-phospho Stat1-PE (4a; BD Biosciences), rabbit anti-mouse Stat1 (9172; Cell Signaling Technology, Danvers, MA), anti-IgG (BD Biosciences), rat anti-mouse IL-18-biotin (93-10C; R&D Systems, Minneapolis, MN), goat anti-mouse IL-18bp-biotin (PeproTech, Rocky Hill, NJ), goat anti-mouse IL-12-biotin (BAF419; R&D Systems), rat anti-mouse IFN-γ-biotin (R4-6A2; BD Biosciences), IgG-biotin (BD Biosciences), anti-rabbit IgG-AlexaFluor 488 (Molecular Probes), and strepavidin-FITC (Biolegend).

Human antibodies used

Antibody-fluorochrome conjugates used were: CD34-FITC (AC136; Miltenyi Biotec, Auburn, CA), CD133/1-APC (AC133; Miltenyi Biotec). Lineage (Lin) markers comprised the following combination of antibody-PE conjugates: CD3 (UCHT1; Biolegend), CD4 (RPA-T4; Biolegend),

CD5 (UCHT2; Biolegend), CD8α (RPA-T8; Biolegend), CD11b (ICRF44; Biolegend), CD19 (HIB19; Biolegend)
