# Supplementary material for: Inhibition of T Cell Protein Tyrosine Phosphatase Enhances Interleukin-18-Dependent Hematopoietic Stem Cell Expansion
Source: Stem Cells. 2012 Nov 8;31(2):293–304. doi: 10.1002/stem.1276 (PMC3593175; doi:10.1002/stem.1276)
Supplement: Supplementary file 6 [file stem0031-0293-SD6.pdf]

Supplemental Table 1. Peripheral blood analysis of the hematopoetic subsets 14, 28 and 56 days post transplants

	% donor GFP^+CD3^+ T cells	% donor GFP^+B220^+ B cells	% donor GFP^+ $Gr\text{-}1^+CD11b^+$ Monocytes & granulocytes
14 days –100 stem cells -- Control	11% ± 2	7% ± 3	76% ± 4
14 days –100 stem cells – TC-PTP inh	10% ± 2	4% ± 2	83% ± 3
14 days –1000 stem cells -- Control	15% ± 3	10% ± 2	69% ± 7
14 days –1000 stem cells – TC-PTP inh	13% ± 5	12% ± 3	70% ± 5
28 days –100 stem cells -- Control	20% ± 4	18% ± 4	62% ± 6
28 days –100 stem cells – TC-PTP inh	19% ± 7	17% ± 4	59% ± 3
28 days –1000 stem cells -- Control	21% ± 5	20% ± 7	54% ± 5
28 days –1000 stem cells – TC-PTP inh	23% ± 3	19% ± 4	52% ± 4
56 days –100 stem cells -- Control	24% ± 6	7% ± 2	62% ± 5
56 days –100 stem cells – TC-PTP inh	25% ± 8	10% ± 4	65% ± 7
56 days –1000 stem cells -- Control	22% ± 5	13% ± 4	60% ± 6
56 days –1000 stem cells – TC-PTP inh	22% ± 4	11% ± 5	67% ± 5
